# Cryptococcal Meningitis in an Apparent Immunocompetent Patient

**DOI:** 10.1177/2324709619834578

**Published:** 2019-04-04

**Authors:** Marian Poley, Richard Koubek, Leonard Walsh, Brian McGillen

**Affiliations:** 1 Penn State Health Milton S. Hershey Medical Center, Hershey, PA, USA

**Keywords:** immunocompetence, cryptococcal meningitis, alcohol abuse

## Abstract

Cryptococcal meningitis is an uncommon and severe infection that tends to affect immunocompromised hosts worldwide and in the United States. Annually it is estimated that there are 200 000 cases of cryptococcal meningitis, with the most recent estimate of 3400 cases per year in the United States alone. However, despite the low incidence, 1-year mortality is estimated at 20% to 30% even with long-term consolidation antifungal therapy. A 37-year-old man presented to the emergency department with headaches, dysarthria, hallucinations, and acute worsening of altered mental status, and he was found to have increased intracranial pressure, cerebrospinal fluid leukocytosis, and few encapsulated yeasts consistent with *Cryptococcus neoformans* meningitis in addition to radiologic evidence consistent with a cryptococcoma of the lungs. This report highlights the occurrence of *Cryptococcus neoformans* meningitis in a presumed immunocompetent host. The clinician should be aware of sources of minor immunosuppression, as they may contribute to development of *Cryptococcus neoformans* meningitis. Mortality in this condition remains high due to subacute presentations and delayed diagnosis in non-immunocompromised patients.

## Introduction

Cryptococcal infection in humans tends to be caused by *Cryptococcus neoformans*, and it is generally found in immunocompromised hosts. Globally, the annual incidence of the cryptococcal disease is approximately 1 000 000 new cases, and over 600 000 patients die with this infection each year. Disseminated infections may certainly occur, but the most severe manifestation of cryptococcal disease is meningitis. It is estimated that 181 000 deaths from cryptococcal meningitis occur annually, with 75% occurring in sub-Saharan Africa. Additionally, the disease accounts for 15% of worldwide AIDS-related deaths. In the United States, estimated that roughly 3400 cases occur per year, with up to 700 deaths.^1^ This case illustrates the need to consider *C neoformans* meningitis, even in patients with apparent normal immune function.

## Case Description

A 37-year-old man presented to the emergency room with symptoms of worsening headaches, dysarthria, and visual and tactile hallucinations in the setting of recent 3-week hospitalization for alcohol detoxification. During the patient’s prior hospitalization at a community hospital in south central Pennsylvania, his course was complicated by seizures, delirium tremens, and acute hypoxic respiratory failure secondary to aspiration pneumonia. The patient was stabilized and was discharged on levetiracetam for seizure prophylaxis. At home, his family reported that his mental status never returned to baseline, and he continued to exhibit visual and tactile hallucinations, as well as worsening episodic dysarthria and drooling, leading to representation.

In terms of the patient’s social history, his wife disclosed that the couple had been married for 4 years and had been in a monogamous relationship to her knowledge. The patient was employed as a tow truck operator and also worked on a farm training horses. He was born in Georgia, resided in Maryland, and traveled to Virginia, Massachusetts, and eastern Canada over the past 3 years. He had no history of intravenous drug use, though the patient had a 30-pack-year cigarette smoking history. He had a history of heavy alcohol use for 20 years. He had not had an alcoholic beverage to her knowledge since his return home from initial hospitalization.

In the emergency department, the patient was febrile to 39.3°C; otherwise vitals were within normal limits. His examination was remarkable for a Glasgow Coma Scale score of 14/15 (verbal response mildly slowed), disorientation to person, place, and date with extreme agitation, and meningismus exacerbated by neck flexion. A complete neurologic examination was unable to be performed, due to mental status, but cranial nerves II to VIII, X, and XI were intact, and the patient had spontaneous movement in all 4 extremities. Laboratory studies revealed a normal basic metabolic panel (Table 1), with the exception of an elevated serum creatinine, a leukocytosis of 18 750 cells/µL, and a negative urine drug screen and blood alcohol level. A non-contrast computed tomography image of the head was negative for acute bleeding, edema, or mass effect. A lumbar puncture (LP) was performed in the emergency department, which showed 130 nucleated cells (81% lymphocytes), protein 76 mg/dL, glucose 15 mg/dL, and an opening pressure of 47 mm Hg (Table 2). Viral serologies—including herpes simplex virus- polymerase chain reaction, Epstein-Barr virus, cytomegalovirus, and varicella-zoster virus—were sent with initial cerebrospinal fluid (CSF) analysis, along with a Venereal Disease Research Laboratory test, *Toxoplasmosis gondii*, and Lyme studies. The patient was started empirically on acyclovir, ceftriaxone, vancomycin, and ampicillin, and was admitted to the Internal Medicine service.

**Table 1.** Basic Laboratory Values From Admission.

Laboratory Test	Patient Value	Normal Range
Sodium	137 mmol/L	136-140 mmol/L
Potassium	4.3 mmol/L	3.5-5.0 mmol/L
Chloride	92 mmol/L	101-111 mmol/L
Bicarbonate	28 mmol/L	20-29 mmol/L
Blood urea nitrogen	12 mg/dL	9-23 mg/dL
Creatinine	0.9 mg/dL	0.8-1.2 mg/dL
Glucose	132 mg/dL	64-100 mg/dL
Calcium	10.4 mg/dL	8.5-10.2 mg/dL
Magnesium	2.5 mg/dL	1.5-2.5 mg/dL
Hemoglobin	14.8 g/dL	13.5-17.5 g/dL (male)
Mean corpuscular volume	100.2 fL	80-96 fL
Platelets	478 k/µL	150-400 k/µL
White blood cells	18.75 k cells/µL	5-10 k cells/µL
Neutrophils, absolute	15.45 k/µL (82.4%)	2.0-7.7 k/µL
Lymphocytes	1.51 k/µL (8.1%)	1.0-3.4 k/µL
Monocytes	1.62 k/µL (8.6%)	0-1.0 k/µL
Basophils	0.03 k/µL (0.2%)	0-0.1 k/µL
Eosinophils	0 k/µL (0%)	0.5 k/µL
Aspartate aminotransferase	33 U/L	0-40 U/L
Alanine aminotransferase	87 U/L	0-31 U/L
Ammonia	19 µmol/L	16-60 µmol/L
Albumin	5.8 g/dL	3.5-5.2 g/dL
Thyroid-stimulating hormone	0.60 UIU/mL	0.30-4.20 UIU/mL
Free T4	1.50 ng/dL	0.9-1.7 ng/dL
Ethanol	<10 mg/dL	<10 mg/dL
Urine drug screen	Nonreactive	Nonreactive
Urinalysis	0-4 RBCs, few bacteria	0-4 RBCs, none

Abbreviation: RBCs, red blood cells.

**Table 2.** Serial Lumbar Puncture Values During Patient’s Hospital Course.

Date	Opening Pressure (cm H_2_O)	Closing Pressure (cm H_2_O)	CSF Removed (mL)
9/5	47	20	N/A
9/7	33	18	10
9/8	28	14	7
9/9	23	15	7
9/10	22	14	7
9/11	20	15	4
9/12	15	N/A	2

Abbreviation: CSF, cerebrospinal fluid.

### Laboratory Testing and Hospital Course

CSF results revealed no bacteria, but few encapsulated yeast identified as *C neoformans.* CSF cryptococcal antigen returned positive at 1:60, prompting discontinuation of empiric antimicrobial therapy and initiation of antifungal therapy (liposomal amphotericin and flucytosine). Serial LPs were performed daily, in accordance with the Infectious Diseases Society of America guidelines for the treatment of cryptococcal meningitis, until opening pressure remained under 20 mm Hg for 2 consecutive days. Computed tomography of the chest, abdomen, and pelvis was suggested to evaluate for other manifestations of cryptococcosis, ultimately disclosing a 1.0-cm right upper lobe ground glass nodularity, along with clustered right middle lobe nodules and linear scarring. HIV and hepatitis C virus antibodies and viral load were checked and were negative. Blood culture was only positive at time of admission, which revealed *C neoformans*.

The patient was maintained on intravenous amphotericin and flucytosine for 26 days. CSF culture for fungal isolates was negative at the 14-day mark while cryptococcal antigen remained elevated at 1:40. His mental and functional status slowly improved. The only complication during his hospitalization was an episode of sinus tachycardia—felt due to paroxysmal sympathetic hypersensitivity as a result of cryptococcal meningitis—which was treated with propranolol. He was transitioned to oral fluconazole on day 27 of treatment, with a goal to complete 56 days (8 weeks) of total therapy. He was ultimately stable to discharge to a rehabilitation facility on hospital day 30.

## Discussion

Invasive cryptococcal disease (cryptococcosis) is caused either by *C neoformans* or *C gattii*.^2,3^ Epidemiologically, *C neoformans* has a worldwide distribution, while *C gattii* is more common in tropical, subtropical, and temperate regions (Australia, South America, Africa, the United States, and Canada).^4^ An important distinguishing feature between the 2 species is the observation that, unlike *C neoformans, C gattii* infection does not tend to opportunistically infect immunosuppressed individuals.^2,5^ Given the above patient infection with *C neoformans*, the following discussion will focus on pathogenesis, diagnosis, and management of *C neoformans*, though the basic principles remain the same with either species.

*Cryptococcus neoformans* is most classically associated with exposure to bird droppings, though the exact relationship between exposure and disease is not clear. Resultant meningitis or meningoencephalitis is thought to result from inhalation of the organism from the environment into the respiratory tract, with hematogenous dissemination to the central nervous system (CNS), possibly with tropism to the CNS by *Cryptococcus*.^3^ The proposed reasoning relates to the fact that *Cryptococcus* species possess multiple virulence factors that allow it to traverse the blood-brain barrier and survive in the relatively nutrient-deplete environment of the CNS.^3^ Entry into the body via respiratory inhalation and ability to grow preferentially in the CNS leads to simultaneous pulmonary and CNS involvement on presentation, noted among multiple case reports on our literature review.^6,7^ Disseminated cryptococcus may involve multiple organ structures, with common sites including skin, lungs, eyes, and CNS, but has also been seen to affect bone and soft tissue.^2,6^

*Cryptococcus neoformans* meningoencephalitis is a rare cause of disease in immunocompetent individuals.^2^ The mechanism by which presumptively immunocompetent hosts contract cryptococcal disease is not fully understood, but illness in these individuals may result from particularly high level of organism exposure, exposure to a cryptococcal strain with increased pathogenicity, or subtle or undetectable immune deficit in the host.^8^ While malignancy, medications, genetic deficits, and AIDS are well-recognized causes of immunosuppression, it is important to consider that several states—notably alcoholism, diabetes mellitus, cirrhosis, and autoimmune conditions—may precipitate mild states of immunosuppression, potentially predisposing hosts to opportunistic infections. In the above-mentioned clinical case, the exact predisposing factor could not fully be determined, but was felt to be due to his prior alcohol abuse. While this patient had reportedly abstained from alcohol for 28 days prior to symptom onset, it has been observed that cryptococcal disease may cause minimal symptoms for up to years prior to reaching full clinical presentation. It is possible that the stress of his prior hospitalization (alcohol withdrawal, pneumonia/respiratory failure requiring intubation) may have triggered worsening of previously occult cryptococcal disease.

Clinical presentation of cryptococcal meningoencephalitis in HIV-negative individuals, according to a multicenter case study involving patients with predisposing risk factors, included headache (73%), constitutional symptoms (fever, weight loss, and night sweats; 68%), and altered mental status (42%) with predisposing factors including corticosteroid therapy (25%), solid organ transplant (15%), chronic organ failure (41%), chronic lung disease (4%), hematologic malignancies (11%), and other malignancies (5%). However, there was no significant predisposing factor reported in 30% of patients with CNS involvement.^9^ Radiographic imaging is frequently unrevealing, though may demonstrate hydrocephalus. HIV-negative patients are, however, more likely to exhibit cryptococcoma and hydrocephalus.^3^ Given previously described tropism to the CNS, meningitis is the most common clinical presentation of cryptococcal disease; however, pulmonary disease may also be present.

Pulmonary cryptococcal disease may be asymptomatic in up to one third of patients and is typically based on radiographic appearance, with the most common finding being pulmonary nodules, as observed in our patient. Masses (cryptocococcoma), cavitation, pleural effusions, and lymphadenopathy may be observed, but are more commonly found in immunocompromised individuals.^6^ A diagnosis of disseminated *Cryptococcus* is made on a positive culture from at least 2 body sites.^2^ Fungemia may be less frequent in immunocompetent patients, though this patient did exhibit one positive blood culture for *C neoformans*.^10^ Patients without apparent immunocompromise are more likely to present with meningitis than those with non-HIV predisposing risk factors, as seen in the above-mentioned patient.^10^ In one study of 35 apparently immunocompetent individuals with CNS cryptococcosis, decreased mentation was observed in 26% of the study population, a prominent feature of this patient’s presentation.^5^ In the same study, overall 49% of the immunocompetent individuals made a complete recovery, with those infected with the *C neoformans* variety *gattii* (serotypes A and D) having a worse prognosis compared with the *C neoformans* variety *neoformans* (serotypes B and C), complete recovery in 38% and 78%, respectively.^5^ In a 2017 multicentered retrospective analysis, higher Glasgow Coma Scale score, CSF leukocyte count >20, and higher CSF glucose levels were associated with better prognosis.^11^ The above-mentioned patient did have CSF leukocyte count over 20, though unfortunately showed relatively slow improvement. It follows that those with immunocompromise in this trial including HIV with CD4 count <200 and malignancy were associated with unfavorable outcome defined as death or survival with relapse or sequelae.^11^ Interestingly, in this analysis, there was no significant difference in outcomes for those with less overt forms of immunocompromise such as cirrhosis, renal disease, alcoholism, diabetes, or immunosuppressive dose of corticosteroids. However, prior studies have found these populations to have poorer outcomes.^11^

As with most CNS infections, primary diagnostic assessment is made on analysis of CSF. A notable finding on LP in cryptococcal meningitis is a marked elevation of opening pressure, which was present in this patient’s case.^3^ A mononuclear predominance in cell count, low glucose, and elevated protein may also be observed. In terms of specific cryptococcal testing, staining with India ink may be insufficient to make a diagnosis, as sensitivity is dependent on fungal burden, which in HIV-infection individuals is 70% to 90%.^3^ CSF culture is considered the gold standard and will be positive in >90% of cases. Cryptococcal antigen testing is important in the early detection of cryptococcal CNS disease. Antigen testing yields rapid results compared with culture, and both latex agglutination assay and ELISA (enzyme-linked immunosorbent assay) testing have sensitivity and specificity >90%, though sensitivity is felt to be lower for non–HIV-infected individuals and should not be used to rule out disease.^3,4^

Management of cryptococcal meningitis is multifaceted, including antifungal therapy and control of intracranial pressure. Most patients will require a prolonged course of antifungal therapy, including induction, consolidation, and maintenance phases. The preferred induction therapy is well-defined as amphotericin B (either liposomal or lipid complex) in combination with flucytosine for at least 2 weeks. If flucytosine is unable to be administered, fluconazole may be used in its place. For HIV-uninfected individuals with neurologic complications (as in this case), induction therapy should be continued for 6 weeks.^3^ For those with poor initial response the duration of induction may be extended.^12^ Assessing response to treatment of immunocompetent hosts differs from immunocompromised hosts in which repeat LP with CSF culture is performed after 2 weeks of induction therapy to assess response in those with known immunocompromised states.^13^ Culture must be used to monitor response to treatment, as cryptococcal antigen testing does not correlate with CSF sterilization at 2 weeks or 180-day outcomes in HIV-negative patients based on previous studies.^14^ Consolidation therapy may be started with fluconazole 400 to 800 mg daily for a period of 8 weeks, followed by maintenance of fluconazole 200 to 400 mg daily for 1 year.^3^ Of note, patients with cryptococcoma require a modified treatment regimen of prolonged antifungal treatment, with consideration of surgical involvement.^15^ Furthermore, with reported development of cryptococcal resistance to fluconazole, a variety of newer antifungal agents and alterative medications are in study.^12^

An aspect of management highlighted in this example is continued monitoring and control of intracranial pressure. Given the high inflammatory burden of cryptococcal meningoencephalitis, there is markedly elevated intracranial pressure. Control of intracranial pressure is important to patient outcomes.^12^ For CSF opening pressures ≥25 cm H_2_O with signs of elevated intracranial pressure during induction therapy, CSF drainage should be performed to target a goal CSF pressure of less than 20 cm H_2_O.^15^ Serial LPs should be performed daily for persistent elevation of CSF pressure until less than 20 cm H_2_O for greater than 2 consecutive days. Temporary percutaneous lumbar drain or ventriculostomy may be indicated in patients requiring recurrent daily drainage. Ventriculo-peritoneal shunt placement may also be considered for elevated intracranial pressures failing conservative management.^15^ Other adjunctive agents such as mannitol, acetazolamide, and glucocorticoids are not supported for lowering intracranial pressure in this setting.^15^

## Conclusion

Cryptococcal meningitis due to *C neoformans* is a relatively rare condition among apparently immunocompetent individuals. When frank immunosuppression is not evident, less-considered sources of minor immunosuppression should be explored. These include, but are not limited to, alcoholism, diabetes mellitus, and chronic liver or kidney disease. Nevertheless, cryptococcal meningitis without a known underlying immunocompromised state carries mortality of 12% and is often delayed in presentation due to the frequently subacute nature of symptom development.^9^ This diagnosis must, therefore, be considered and pursued in any patient with signs and symptoms of meningitis, lymphocytic CSF findings, and elevated opening pressure. Prompt initiation of therapy includes antifungal agents and control of intracranial pressure to avoid morbidity and mortality, as well as controlling underlying causes of potential immunosuppression.
